# Book Reviews

**Published:** 1907-10

**Authors:** 


### BOOK REVIEWS.

THE PRINCIPLES AND PRACTICE OF MODERN SURGERY. By Roswell Park. M. D.., Professor of Surgery in the University of Buffalo, Buffalo, N. Y. In one very handsome imperial octavo volume of 1072 pages, with 722 engravings and 60 full-page plates in colors and monochrome. Cloth, $7.00, net; leather, $8.00,net. Lea Brothers & Co., Philadelphia and New york, 1907.

The sign of a great and growing subject is found in its literature, the measure of its activity and growth. This is particularly applicable to surgery, endless and unresting in its advance and possibilities. However, no justification is necessary in the case of a work from the pen of so eminent and mature a surgeon as Professor Park. He is peculiarily qualified upon both main divisions, as always an enthusiastic worker in surgical pathology, and as an equally resourceful and successful pratitioner of the art. Thus he has been able to produce a book which is well balanced, complete, and with all its information well interrelated. His skill as a teacher of the first rank is manifest in his orderly arrangement and clear exposition. Accordingly, his work possesses a wide range of importance, for it affords the student a logical training, thereby minimizing the labor both for him and his teacher, and serves the general pratitioner and surgeon equally as well as an authoriative guide in the most directly responsible branch of all professional work.

This new individual book is the successor of the Surgery by American Authors edited by Professor Park, which ran through three editions. His collaborators therein have most willingly placed their work and accompanying illustrations at his service. As Professor Park is equally at home in the surgical literature in English, German and French, the three languages to which everything in the civilized world must come for dissemination, his Modern Surgery may be trusted as an authoritative exposition of the world's most advanced views and practice at the present time.

---

A TREATISE ON FRACTURES AND DISLOCATIONS. By Lewis A. Stimson, B. A., M. D., Professor of Surgery in Cornell University Medical College, New York. New (5th) edition, thoroughly revised. Octavo, 847 pages, with 352 engravings and 52 plates. Cloth, $5.00, net; leather, $6.00, net, half morocco, $6.50, net. Lea Brothers & Co., Philadelphia and New York, 1907.

The universality of these injuries, and their demand for prompt treatment on the spot, require all general practitioners as well as surgeons to be conversant with the best manipulations and management. Their variety is too great for full consideration in works on general surgery, and the result of partial knowledge eventuating in a shortened leg or a stiff joint is apt to be a law suit damaging to reputation and pocket. It behooves every medical man to be prepared.

In his volume, which has now become classical and the authority accepted both by the profession and the courts, Dr. Stimson has covered every known form of these lesions, many of which were first described in his pages. His literary style is notable for clearness so that his readers need not err. His work stands alone in literature as including a full consideration of Dislocations as well as Fractures, two cognate subjects advantageously handled in close connection. This single volume accordingly affords complete and authoritative information on a large and important surgical specialty. Its recognized position is shown by the demand for another new edition, the fifth, which the author has again revised to the latest date.

---

MANUAL OF DISEASES OF THE EYE. By Charles H. May Chief of Clinic and Instructor in Ophthalmology, College of Physicians and Surgeons, Medical Department, Columbia University. New York.—1890-1903; Ophthalmic Surgeon to the City Hospitals, Randall's Island. New York; Consulting Ophthalmologist to the French Hospital, to the Gouverneur Hospital, and to the Red Cross Hospital, New York; Adjunct Ophthulmic Surgeon to Mt. Sinia Hospital, New York, etc. Fifth edition revised with 362 original illustrations, with 22 plates. with 63 colored figures. Price $2.00, net. Wm. Wood & Co.

The excellence of this work is attested by the promptness with which each editor is exhausted and another demanded. It is an ideal book for the student and practitioner.

---

A TEXT-BOOK OR PRACTICAL DIAGONSIS. The Use of Symptoms in the Diagnosis of Disease. By Hobart Amory Hare, M. D., Professor of Therapeutics in the Jefferson Medical College of Philadelpha. New (6th) edition, thoroughly revised and written. Octavo, 616 pages, with 203 engravings and 16 full-page plates. Cloth, $4.50, net; leather, $5.50, net Lea Brothers & Co., Philadelphia and New York, 1907.

Professor Hare is as resourceful in his literary methods as in practice, and in his Diagnosis he has produced a work which must have taxed his ingenuity and industry, but he has made a straight and smooth path for his readers. That they have been prompt and steadfast in appreciation is shown by the call for six editions.

The plan of the work is exactly the reverse of the usual book on Diagnosis, which analyzes disease into symptoms and requires the reader to recombine them when meeting a case. Dr. Hare's method might be termed the natural way, as he approaches his subject as the physcian must approach his patient, namely, symptoms first, and upbuilds his diagnosis on these units. Thus the discovery of any marked symptom, such as vomiting, leads the reader to the point where its diagnostic significance is discussed and the differentiation of the various conditions in which it may occur. The whole field is covered in this convenient way. Instructive and typical engravings and plates are liberally employed. The revision for this new edition has been most thorough, bringing the volume well up to the latest knowledge.

As indicating the popularity of Professor Hare's works, it is worthy of note that within the past few months have appeared this sixth edition of his Diagnosis, the twelfth edition of his Therapeutics, and the second of his Practice, the last named having run through two very large printings of its first edition and into the second in two years. Such a record would be difficult to parallel.

